# Supplementary material for: Sequence-based generative AI design of versatile tryptophan synthases
Source: Nat Commun. 2026 Jan 14;17:1680. doi: 10.1038/s41467-026-68384-6 (PMC12909874; doi:10.1038/s41467-026-68384-6)
Supplement: Supplementary file 1 — Supplementary Information [file 41467_2026_68384_MOESM1_ESM.pdf]

Supplementary Materials for

Sequence-Based Generative AI Design of Versatile Tryptophan Synthases

Théophile Lambert[1,2], Amin Tavakoli[3], Gautham Dharuman[4], Jason Yang[1], Vignesh Bhethanabotla[1], Sukhvinder Kaur[5], Matthew Hill[5], Arvind Ramanathan[4], Anima Anandkumar[3,*], and Frances H. Arnold[1,*]

[1]Division of Chemistry and Chemical Engineering, California Institute of Technology, Pasadena, CA, USA
[2]Permanent address: Université Paris-Saclay, CNRS UMR8182, Institut de Chimie Moléculaire et des Matériaux d'Orsay (ICMMO), 17 Avenue des Sciences, 91400 Orsay, France
[3]Department of Computing and Mathematical Sciences, California Institute of Technology, Pasadena, CA, USA
[4]Argonne National Laboratory, Lemont, IL, USA
[5]Elegen Corp, 1300 Industrial Road #16, San Carlos, CA, USA
[*]Corresponding authors: anima@caltech.edu, frances@cheme.caltech.edu

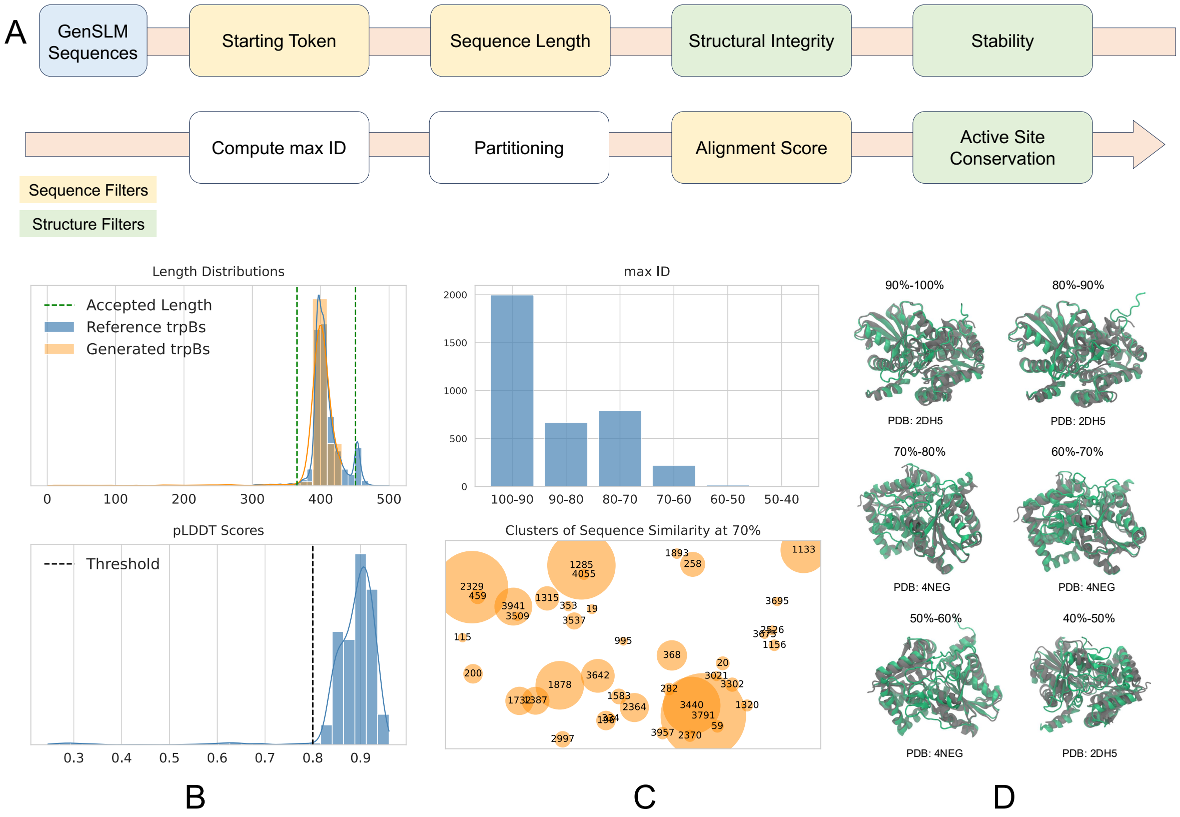

Figure S1: **Filtering of the generated *trpB* sequences**. (A) A schematic depiction of our proposed filtering process. (B) Distributions of length and pLDDT scores. (C) The sequence identity and sequence similarity of the generated *trpB* sequences. (D) The predicted structure of six selected samples from each partition of the max ID aligned with their closest match from the references.

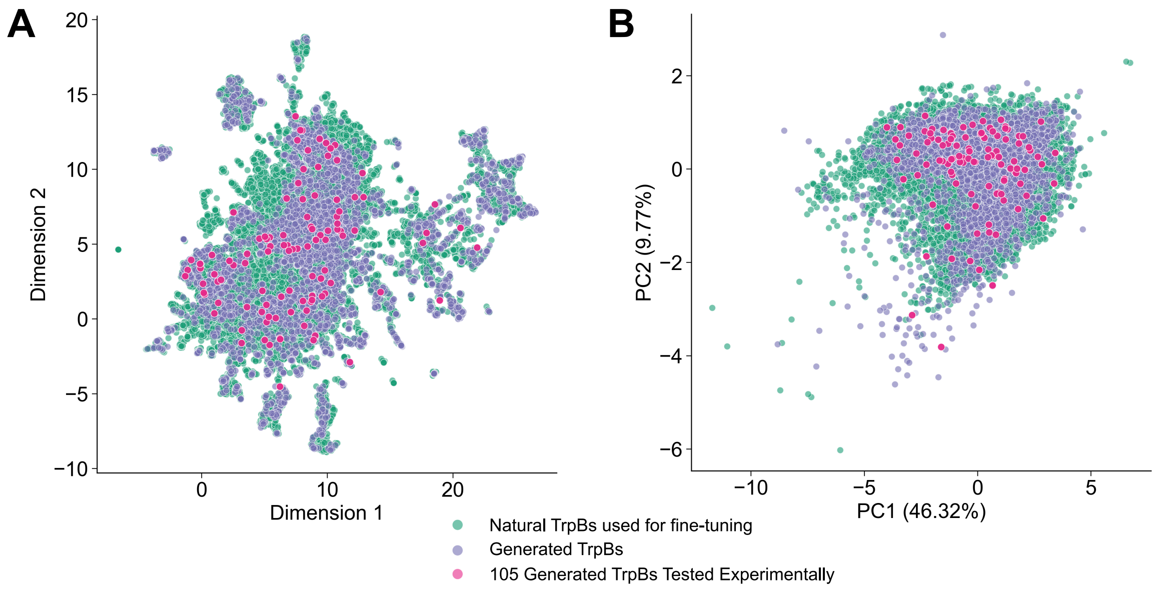

Figure S2: **2D visualizations of the GenSLM embeddings.** (A) UMAP projection of natural and GenSLM-TrpB sequences. (B) PCA projection of the same dataset.

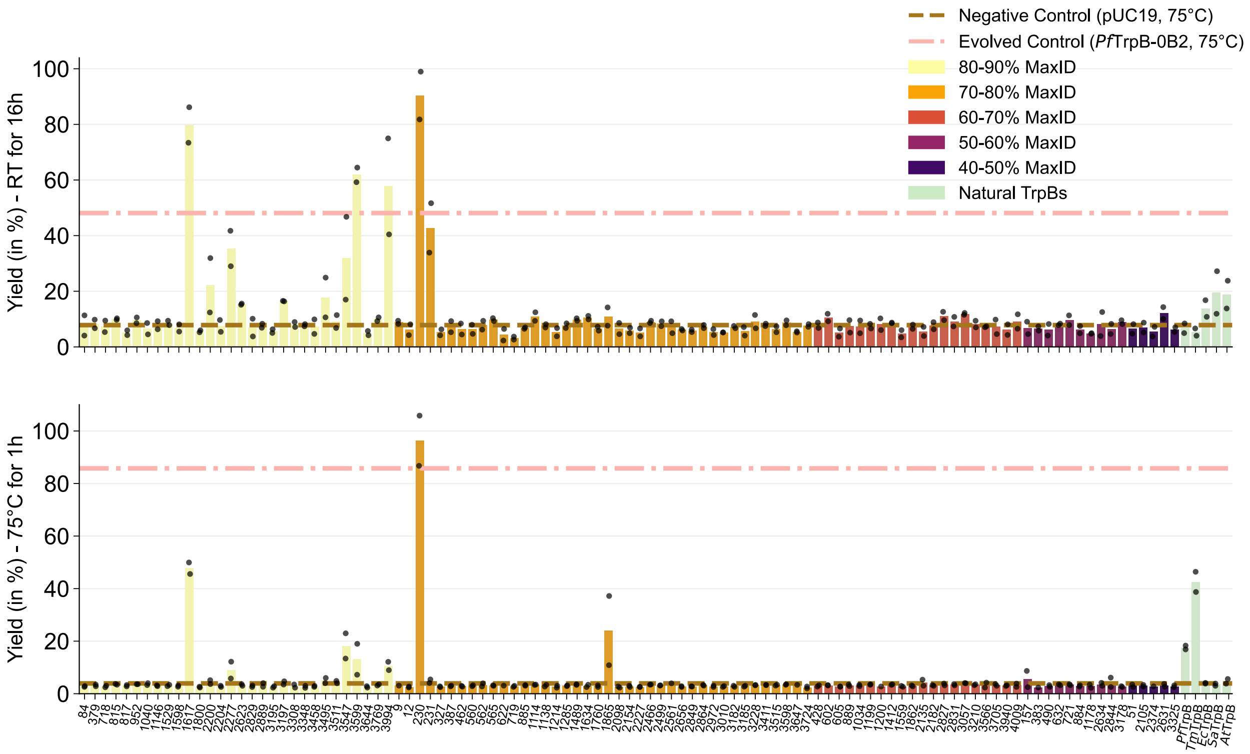

Figure S3: **Tryptophan yields of GenSLM-TrpBs and natural TrpBs.** Yields are measured after 16 h at room temperature and 1 h at 75 °C and represent the mean of biological duplicates ($n = 2$). MaxID: maximum sequence identity to natural TrpBs. Source data are provided as a Source Data file.

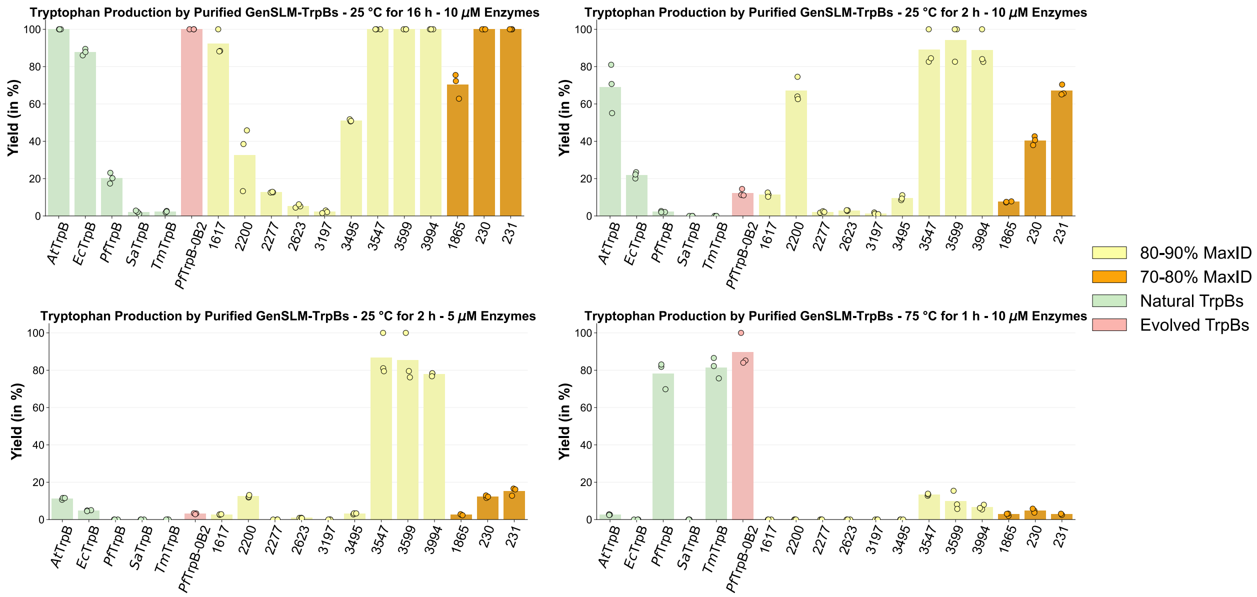

Figure S4: **Tryptophan formation catalyzed by purified GenSLM-TrpBs.** Product yields are measured under varying protein concentrations, reaction times, and temperatures. Bar heights represent the mean of technical triplicates ($n = 3$). MaxID: maximum sequence identity to natural TrpBs.

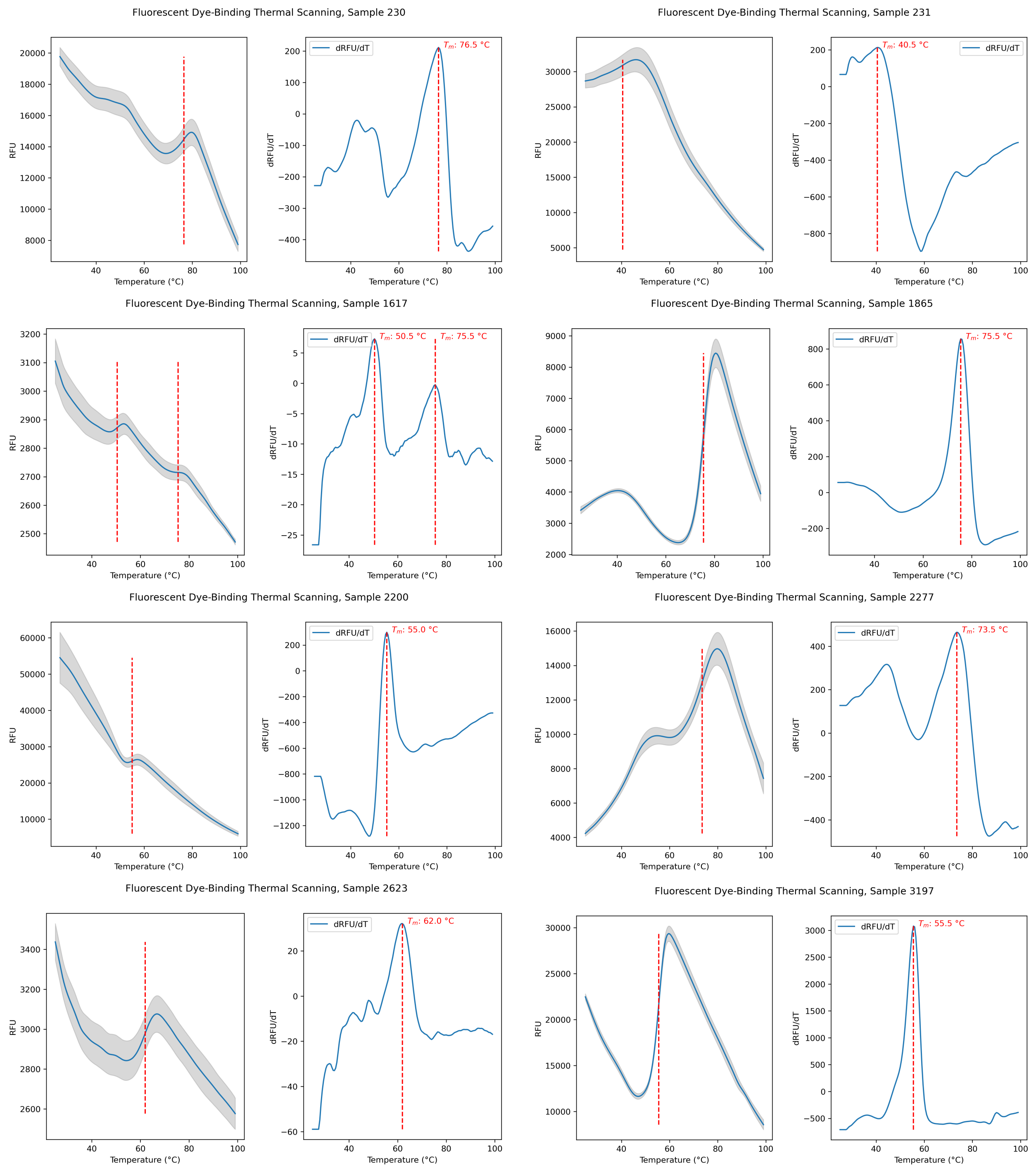

Figure S5: **Stability assays of purified GenSLM-TrpBs** Melting temperatures were determined by thermoshift assay using purified GenSLM-TrpBs. Raw fluorescence and first-derivative traces are shown in relative fluorescence units (RFU). Curves represent the mean of technical triplicates ($n = 3$), with shaded regions indicating the standard deviation.

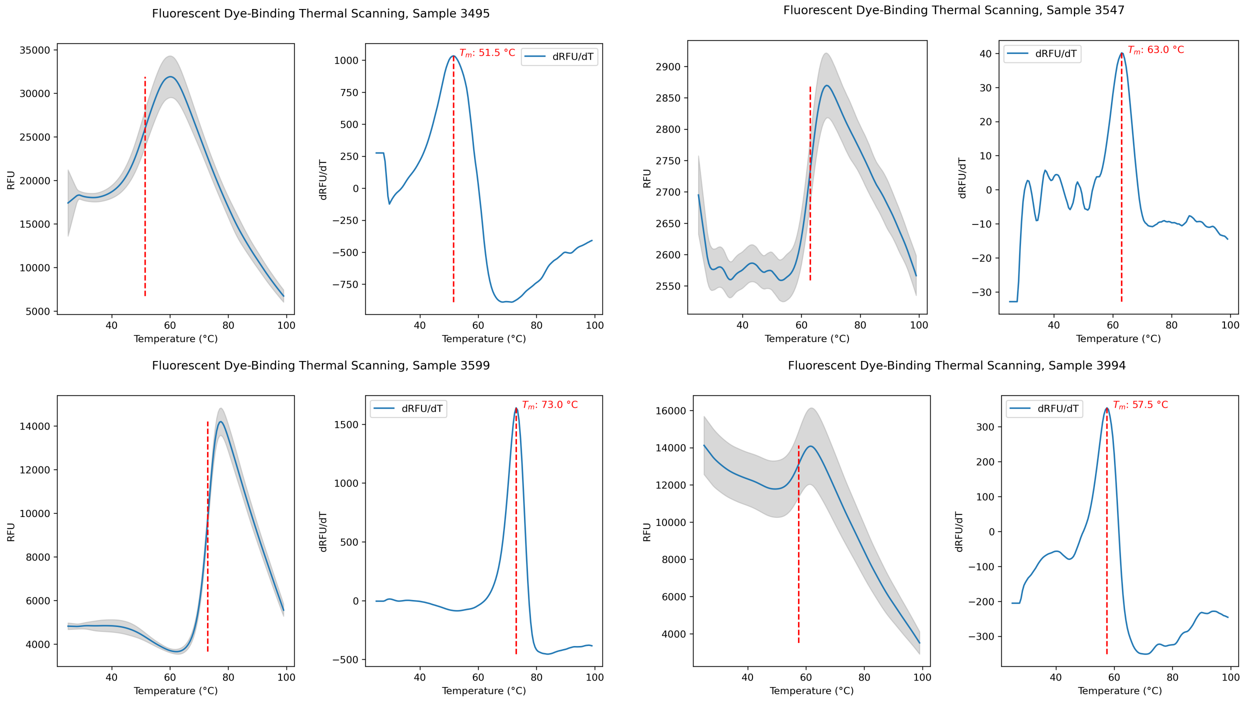

Figure S5: (continued): **Stability assays of purified GenSLM-TrpBs** Melting temperatures were determined by thermoshift assay using purified GenSLM-TrpBs. Raw fluorescence and first-derivative traces are shown in relative fluorescence units (RFU). Curves represent the mean of technical triplicates ($n = 3$), with shaded regions indicating the standard deviation.

L-ser + naphtol, **TmTyrS6**

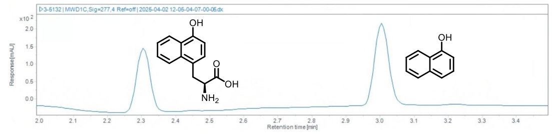

L-ser + 4-NO_2-indole, **Pf2A6**

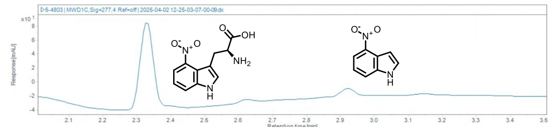

L-ser + 5-NO_2-indole, **Pf2B9**

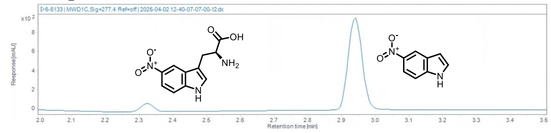

L-ser + 6-CN-indole, **Pf0A9**

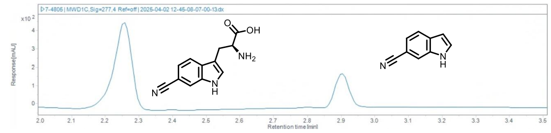

7-CN-indole, direct injection

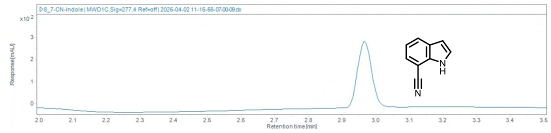

L-ser + 7-CN-indole, **Pf2A6**

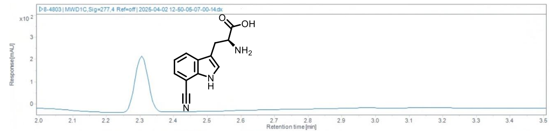

L-thr + indole, **Pf2B9**

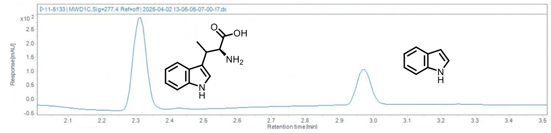

L-ser + 5-F-indole, **TmTriple**

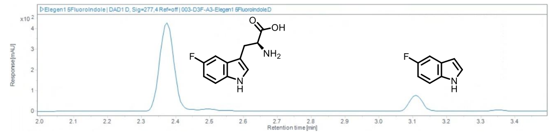

Figure S6: **HPLC traces for reactions with various non-canonical substrates catalyzed by previously engineered TrpBs.** Chromatograms showing the UV channel at 277 nm corresponding to the reaction product obtained using engineered TrpB variants, in bold, and the indicated substrates. Peaks corresponding to the reaction products and the remaining indole derivative, if present, are shown, while L-serine and L-threonine are not visible under these chromatographic conditions, as they elute at the dead volume of the column.

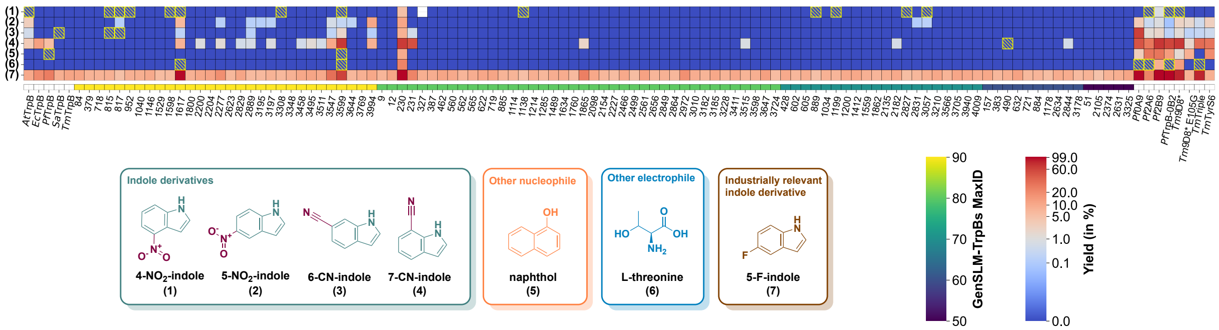

Figure S7: **Reaction yields of GenSLM-TrpBs with non-natural substrates.** Yields are estimated from absorbance at the isosbestic point (277 nm). Yellow dashed boxes denote reactions where product formation was confirmed by mass spectrometry above background levels, but remained below the UV detection threshold. Yields are displayed using a power-law normalization with $\gamma = 0.15$. Source data are provided as a Source Data file.

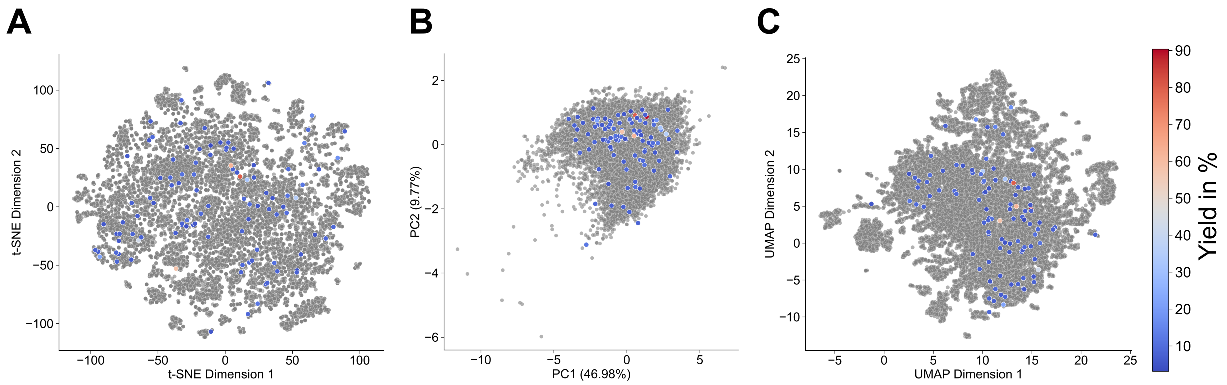

Figure S8: **2D visualizations of the GenSLM embeddings, colored by yield at room temperature (16 h) of tested variants.** The yield values correspond to the room temperature tryptophan yields reported in Fig. S3. (A) t-SNE projection. (B) PCA projection. (C) UMAP projection. Grey dots correspond to the natural TrpBs used for fine-tuning. Active GenSLM-TrpBs are distributed throughout the natural sequence space, indicating that the model did not converge to a single solution.

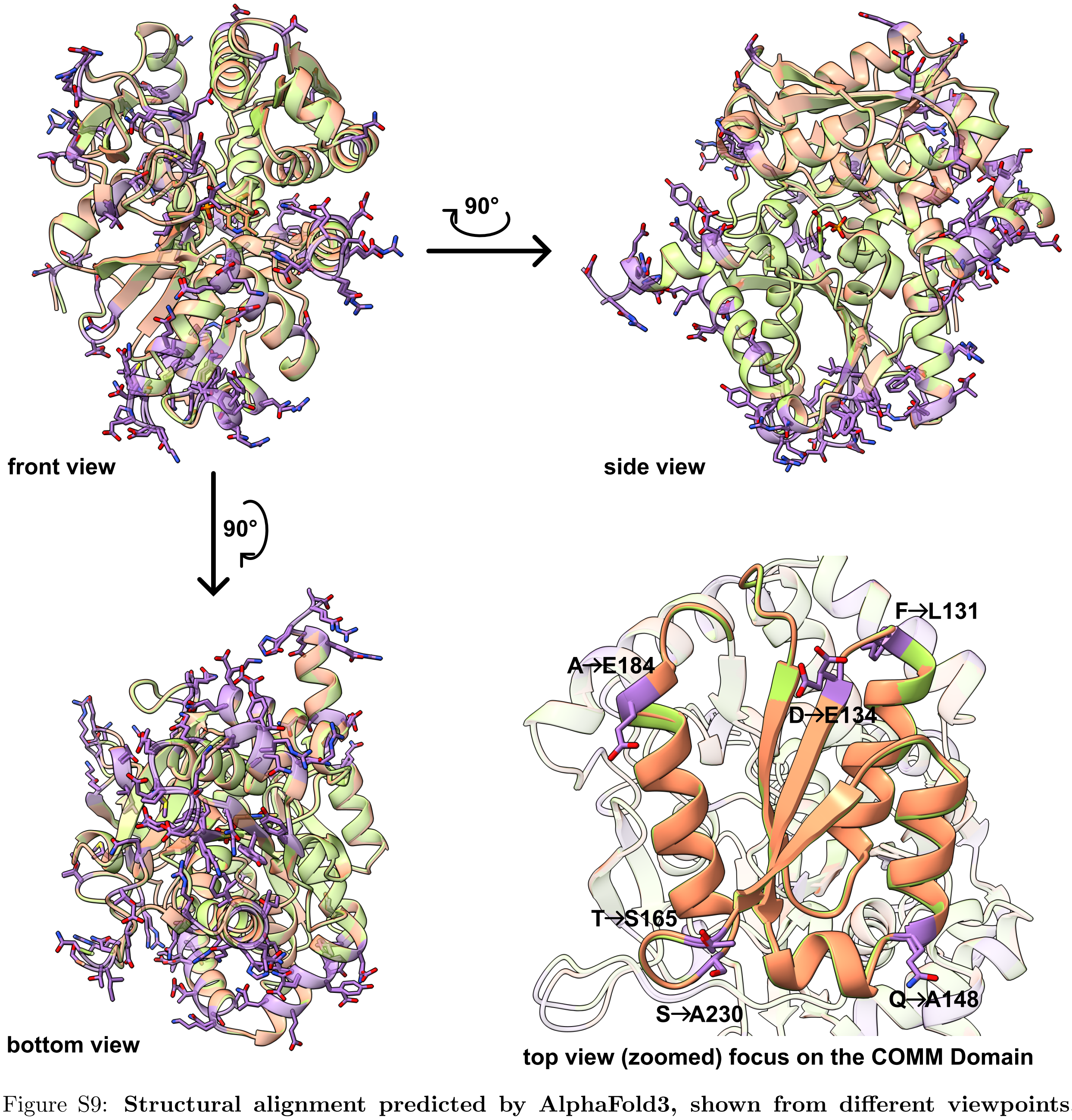

Figure S9: **Structural alignment predicted by AlphaFold3, shown from different viewpoints.** **230** is shown in orange, *Nd*TrpB in green, and non-conserved residues are highlighted in purple. All non-conserved residues are displayed, and the close-up depict the COMM domain, showing six mutations between the two structures. The mutation are indicated from **230** to *Nd*TrpB.

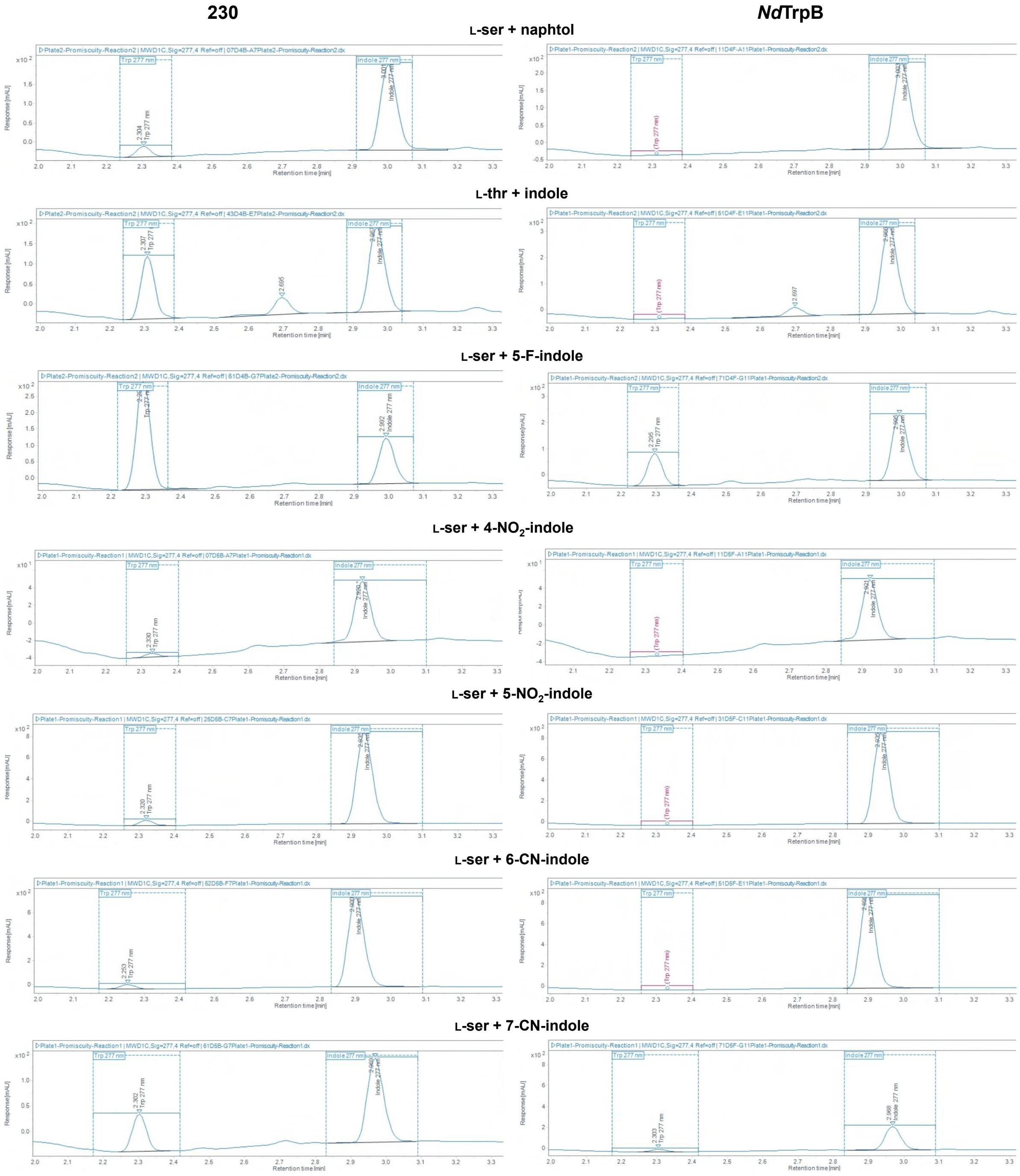

Figure S10: **HPLC traces for reactions with various non-canonical substrates catalyzed by 230 and *Nd*TrpB.** Chromatograms showing the UV channel at 277 nm corresponding to the reaction product obtained from **230** and *Nd*TrpB using the indicated substrates. The signal labeled Trp 277 nm corresponds to the integrated product peak, while the signal labeled Indole 277 nm corresponds to the integrated remaining indole derivatives.
