# Supplementary material for: Sequence-based generative AI design of versatile tryptophan synthases
Source: Nat Commun. 2026 Jan 14;17:1680. doi: 10.1038/s41467-026-68384-6 (PMC12909874; doi:10.1038/s41467-026-68384-6)
Supplement: Supplementary file 4 — Description of Additional Supplementary Files [file 41467_2026_68384_MOESM4_ESM.pdf]

<u>Description of Additional Supplementary Files</u>

Supplementary Data 1.

Description: Sequences of all natural, evolved, and generated TrpBs used in this study, along with their sources.
